# The Eye As a Biomarker for Alzheimer's Disease

**DOI:** 10.3389/fnins.2016.00536

**Published:** 2016-11-17

**Authors:** Jeremiah K. H. Lim, Qiao-Xin Li, Zheng He, Algis J. Vingrys, Vickie H. Y. Wong, Nicolas Currier, Jamie Mullen, Bang V. Bui, Christine T. O. Nguyen

**Affiliations:** ^1^Department of Optometry and Vision Sciences, University of MelbourneMelbourne, VIC, Australia; ^2^Melbourne Brain Centre, The Florey Institute of Neuroscience and Mental HealthParkville, VIC, Australia; ^3^Biogen Inc.Cambridge, MA, USA; ^4^AstraZeneca NeuroscienceCambridge, MA, USA

**Keywords:** biomarker, Alzheimer's disease, neurodegeneration, ocular, eye, retina

## Abstract

Alzheimer's disease (AD) is a progressive neurodegenerative disorder resulting in dementia and eventual death. It is the leading cause of dementia and the number of cases are projected to rise in the next few decades. Pathological hallmarks of AD include the presence of hyperphosphorylated tau and amyloid protein deposition. Currently, these pathological biomarkers are detected either through cerebrospinal fluid analysis, brain imaging or post-mortem. Though effective, these methods are not widely available due to issues such as the difficulty in acquiring samples, lack of infrastructure or high cost. Given that the eye possesses clear optics and shares many neural and vascular similarities to the brain, it offers a direct window to cerebral pathology. These unique characteristics lend itself to being a relatively inexpensive biomarker for AD which carries the potential for wide implementation. The development of ocular biomarkers can have far implications in the discovery of treatments which can improve the quality of lives of patients. In this review, we consider the current evidence for ocular biomarkers in AD and explore potential future avenues of research in this area.

## Introduction

**Figure 1:**
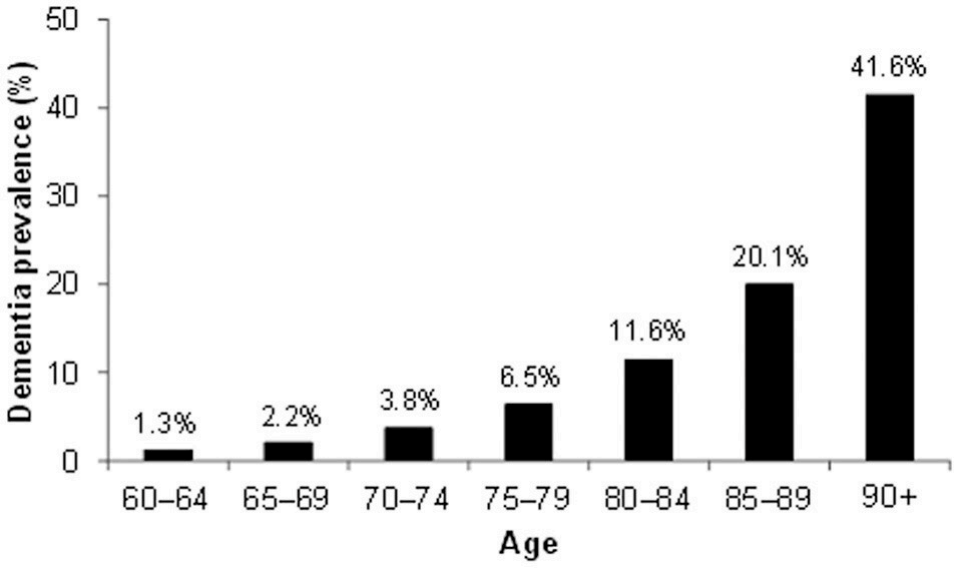
**Dementia world prevalence by age**. Data based on meta-analyzed estimates of dementia prevalence (%) generated from Poisson random effects models from WHO (2012).

Alzheimer's disease (AD) is a chronic, progressive neurodegenerative disease leading to severe cognitive loss and eventual death. Of the dementias, AD is the most common, accounting for between 60 and 70% of all dementias, depending on geography (WHO, 2012). Cohort studies around the world show that the number of people with AD increases with age, with roughly 1 in 5 people suffering from AD by the age of 85 (Figure 1).

**Figure 2:**
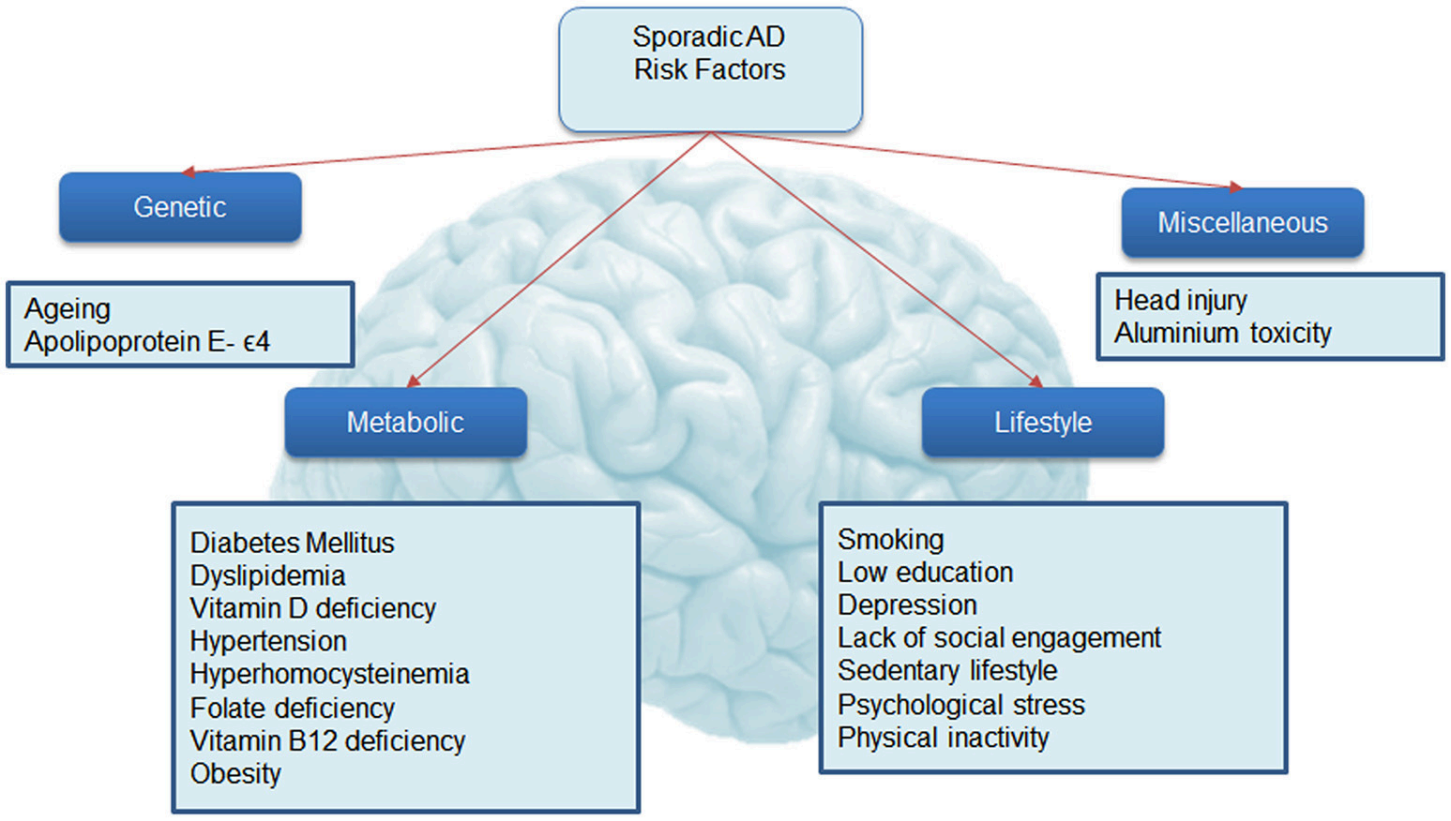
**Risk factors associated with Alzheimer's disease**. This figure shows known associations with Alzheimer's disease.

The risk of developing the disease with age doubles every 5.9 years from 3.1 per 1000 persons aged 60–64 to 175 persons per 1000 at age 95+ (WHO, 2012), making age the strongest risk factor for AD. Characteristic to the disease is the profound atrophy of the brain accompanied by amyloid-beta (Aβ) plaques and the presence of tau neurofibrillary tangles (NFTs). Besides aging, genetics play a major role in the development of sporadic AD. Carriers of the apolipoprotein E (APOE) ϵ4 allele (13.7% of the world's population) face an increased risk of developing the disease (Farrer et al., 1997), depending on whether they have one or two copies of the ϵ4 allele. The estimated lifetime risk of developing the disease is higher in women with one (30%) or two (60%) copies of the ϵ4 allele, compared with men (23 and 51% respectively, Genin et al., 2011). Environmental factors for the disease (Figure 2) include diabetes mellitus, midlife hypertension, present smoking, depression, cognitive inactivity, physical inactivity, obesity, level of education, lack of social engagement (Flicker, 2010; Barnes and Yaffe, 2011). These modifiable factors (Table 1) carry varying degrees of risk and in combination may account for up to half of the AD cases worldwide (Barnes and Yaffe, 2011).

**Table 1:** **Risk factors for sporadic Alzheimer's disease**.

**Risk factor**	**Relative risk**
Lack of social engagement	2.34 (1.18–4.65)
Depression	1.90 (1.55–2.33)
Physical inactivity	1.82 (1.19–2.45)
Hypertension (midlife)	1.61 (1.16–2.24)
Obesity (midlife)	1.60 (1.34–1.92)
Smoking	1.59 (1.15–2.20)
Low education	1.59 (1.35–1.86)
Diabetes	1.39 (1.17–1.66)

This table shows the most commonly identified modifiable risk factors in non-familial AD and associated relative risk.

**Figure 3:**
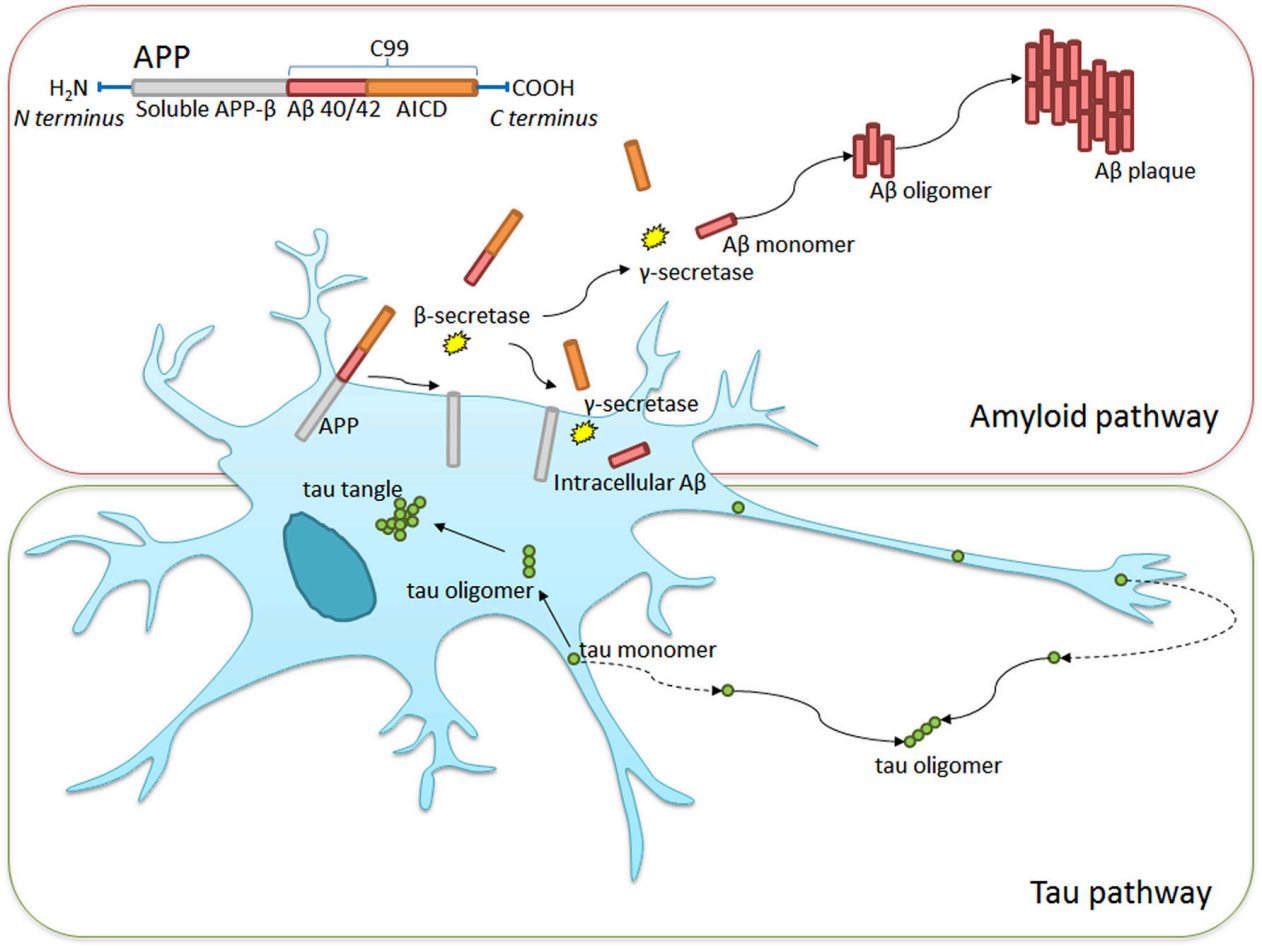
**Intra- and extra-cellular Alzheimer's disease hallmark formation**. Amyloid precursor protein (APP) transmembrane protein contains between 365 and 770 amino acids beginning with the N- and ending with the C-terminus. β-secretase cleavage leads to the formation of a 99-chain amino acid at the C terminus (C99). It undergoes further cleavage via γ-secretase to form either Aβ-40 or Aβ-42 monomers. These monomers clump together, taking on complex formations eventually leading to Aβ plaque formation. Similarly, tau monomers clump to form complex oligomers and eventual neurofibrillary tangles, though this process is less well understood. Non-pathological APP processing via α-secretase is not shown in the diagram.

At present, physicians make the diagnosis of AD when patients already exhibit early cognitive losses. The diagnosis is formalized through mental state or cognitive examinations, alongside vascular and neurological assessments to rule out other causes (Burns and Iliffe, 2009). These tests signify the beginning of the irreversible process leading to dementia. The diagnosis is only confirmed *post mortem* though an examination of the brain. At present, the average survival from diagnosis to death is 4.6 years, affording little opportunity for treatment outside of palliative care. Figure 3 summarizes the mechanisms leading to the formation of Aβ plaques and tau tangles, both of which are hallmarks of the condition.

Significant advances have been made in the development of *ante mortem* diagnostic tools or biomarkers for the disease. Biomarkers or “surrogate measures” of a disease are useful because they enable early diagnosis. Moreover, a good biomarker also enables assessment of drug efficacy both in the laboratory and in the clinic. Tools that can reliably triage drugs that are worth taking forward into progressively more expensive Phase I, II, and III clinical trial phases would considerably reduce the cost of drug development. Techniques that are simple, non-invasive, quantitative and objective lend themselves well to being biomarkers for preclinical and clinical trials. There is a growing need for a biomarker in Alzheimer's disease as recent clinical findings suggest that successful treatment needs to start in the prodromal stages of the disease (Ising et al., 2015). How such prodromal stages can be identified is thus of critical importance.

At present, the most well established biomarkers include those found in cerebrospinal fluid (CSF) (Aβ-42, T-tau, p-tau) and in the brain (fluorodeoxyglucose [FDG]- and Pittsburg Compound B [PiB]- Positron Emission Tomography (PET) with reported sensitivities and specificities of about 0.8 (Rabinovici et al., 2011; Ferreira et al., 2014). Whilst these methods have considerably advanced our understanding of the disease, clinically they fall short of the criteria necessary for large-scale population screening. Such methods can also be expensive, require repeat exposure to radiation (PET imaging) or are invasive (lumbar puncture to obtain CSF sample). The search for AD biomarkers has expanded to include other forms of brain imaging such as near infrared and brain volume scans (Hoffman et al., 2000; Csernansky et al., 2004; Klunk et al., 2004; Hintersteiner et al., 2005), as well as assays of, blood (Koyama et al., 2012), skin (Khan and Alkon, 2010; Khan et al., 2015), urine (Ghanbari et al., 1998), odor (Kimball et al., 2016), and olfactory deficits (Devanand et al., 2000; Tabert et al., 2005).

**Figure 4:**
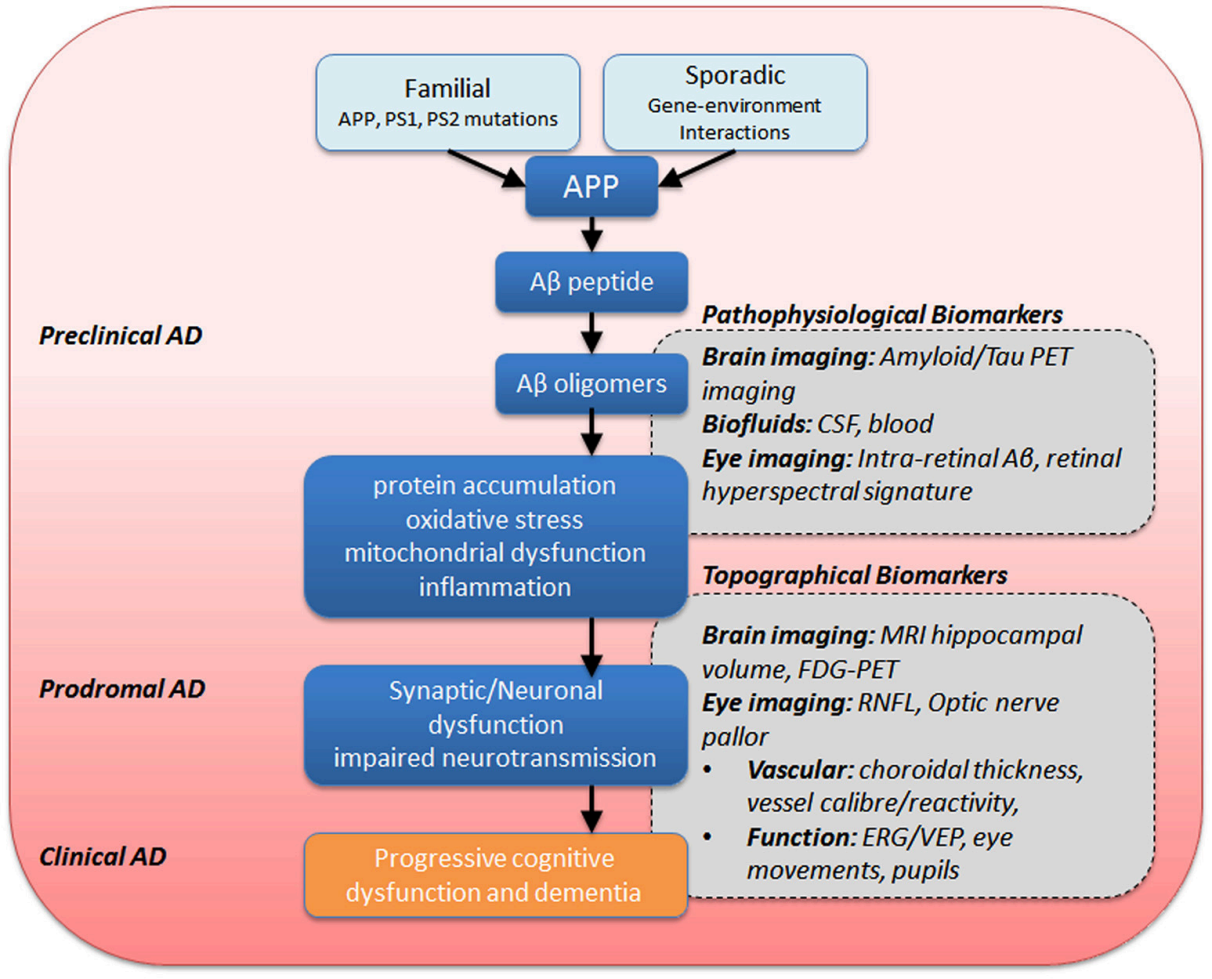
**Summary of eye and brain biomarkers of AD**. APP, amyloid precursor protein; PS1/PS2, presenilin-1 −2, ERG, electroretinography; CSF, cerebrospinal fluid; MRI, magnetic resonance imaging; PET, positron emission tomography; FDG-PET, fluorodeoxyglucose-PET; RNFL, retinal nerve fiber layer; VEP, visual evoked potential.

Given that many Alzheimer's sufferers report visual symptoms (Schlotterer et al., 1984; Sadun et al., 1987; Cronin-Golomb et al., 1993), there has been an increased interest in potential ocular biomarkers. Indeed, there have been reports that some visual symptoms can precede the onset of dementia, and have been attributed to the development of senile plaques and tangles in the visual regions of the brain (Mentis et al., 1996; McKee et al., 2006; Brewer and Barton, 2014). In addition, a visual variant of AD (VVAD) affecting relatively younger persons has been identified, though it is important to distinguish this as a separate pathophysiological entity known as posterior cortical atrophy. Patients suffering from VVAD typically first present with visual symptoms in the fifth or sixth decade of life and eventually the cognitive decline follows the course more typically seen in AD (Levine et al., 1993; Lee and Martin, 2004; Kaeser et al., 2015). In addition to potentially important early visual changes, the eye is very accessible and the retina can be easily imaged, thus making ocular biomarkers attractive. Figure 4 summarizes the mechanisms thought to be involved in AD. Along this sequence of pathological changes are opportunities for various biomarkers including those involving the eye. These will be discussed in greater detail below.

## Retinal biomarkers

### Retinal nerve fiber layer and optic nerve

The first histological evidence for retinal abnormalities in AD was presented by Hinton et al. (1986) who found widespread ganglion cell losses (the output neurons of the eye), retinal nerve fiber layer thinning (RNFL, ganglion cell axons) and optic nerve degeneration in AD versus healthy controls. This finding was supported by further studies (Blanks et al., 1996) and provides an anatomical basis for the use of retinal imaging as a potential marker of AD. Conventional fundus photography was met with limited success due to poor nerve fiber resolution (Tsai et al., 1991; Hedges et al., 1996).

The advent of commercially available, high resolution imaging modalities such as confocal scanning laser ophthalmoscopy (SLO) and optical coherence tomography (OCT) allow for repeatable *in vivo* quantification of optic nerve topography and nerve fiber layer. Scanning laser ophthalmoscopy, has the advantage over conventional photographs of the back of the eye in that it employs a confocal scanning laser system, thus allowing for fine depth resolution. Using SLO, Kergoat et al. (2001) and Danesh-Meyer et al. (2006) demonstrated RNFL thinning in the macula and peripapillary regions of the retina. Optical coherence tomography uses low coherence interferometry to detect small differences in *in vivo* refractive indices, thus returning high resolution cross sections of the retina. Using OCT, Berisha et al. (2007) showed overall RNFL thinning in AD, with preference for superior RNFL loss. This finding is corroborated by a number of other studies (Lu et al., 2010; Kesler et al., 2011; Kirbas et al., 2013; Cheung et al., 2015). Cheung et al. (2015) in a large cohort of AD and patients with mild cognitive impairment (MCI) showed that the ganglion cell complex (which includes the RNFL, ganglion cell bodies and their dendrites) at the macula (where this anatomical feature has the best signal to noise characteristics) is a more sensitive indicator of the disease than RNFL thickness. Using this method, they were able to distinguish AD and MCI from healthy controls after adjusting for age, gender, ethnicity and OCT signal strength.

A challenge in using the macular ganglion cell complex or RNFL as biomarkers is its specificity, which is prone to confounds introduced by aging and other co-existing pathologies such as glaucoma. Perhaps one way to disambiguate age-related changes is to assess much wider areas of retina. Age-related changes are thought to be much more generalized, whereas there is some evidence for more sectoral losses in diseases such as AD (Parisi et al., 2001; Berisha et al., 2007; Kesler et al., 2011) and glaucoma. Whether AD and glaucoma exhibit mutually exclusive patterns of loss remains to be seen but the recent advent of wide field spectral domain OCT (Heidelberg Engineering, 2016) will increase our capacity to topographically map ganglion cell complex and RNFL changes.

As we learn more about the sequence of events and the neurodegenerative changes in AD there may be further opportunities for structural retinal biomarkers. For example, there is evidence that synapse loss predates neuronal loss and that the remaining neurons become less well connected to their synaptic partners. This may account for why synaptic density is the best correlate of cognitive decline in AD (DeKosky and Scheff, 1990; Scheff et al., 1990, 1993, 2007; Terry et al., 1991; Masliah et al., 1994; Ingelsson et al., 2004), which may also explain why the macular ganglion cell complex is more sensitive to MCI than RNFL thickness as it is inclusive of the inner plexiform layer containing dendrites of and synaptic connections between retinal ganglion cells and bipolar cells. In addition to synaptic loss, early change to neurons includes the disruption of microtubules (Matsuyama and Jarvik, 1989; Mandelkow et al., 2004; Gasparini et al., 2011). Ganglion cell axons in the RNFL contain microtubules, which give rise to the property of birefringence, whereby their refractive index depends on the polarization of the incident light. Thus, retinal imaging based on the projection of polarized light is extremely sensitive to the loss of microtubules in ganglion cells. Reductions in the birefringence signal as measured using scanning laser polarimetry (GDx) has been shown to precede RNFL thinning as measured by OCT (Huang et al., 2006; Fortune et al., 2008). Polarization sensitive OCT (PS-OCT) is a rapidly developing imaging modality that may have substantial implications for retinal imaging in AD (Pircher et al., 2011). By combining multiple techniques including wider topographical mapping, comparing inner plexiform and RNFL thickness and birefringence it may be possible to return certain signatures of loss for aging, glaucoma and AD thus improving specificity for retinal imaging biomarkers of AD.

### Retinal blood flow and vasculature

The most commonly reported vascular problems in AD include impaired Aβ clearance, blood-brain-barrier compromise, reduced blood vessel density, smaller vessel diameter (vasoconstriction) and reduced blood flow (Zlokovic, 2011). Given similarities between blood vessels in the brain and eye (for review, see Patton et al., 2005), investigators have considered the potential usefulness of quantifying the blood vessels in the eye as a biomarker for AD. Berisha et al. (2007) in a small cohort showed a significant narrowing of retinal veins and reduced venous blood flow in AD compared with healthy controls using a laser Doppler imaging device. Using similar methodology, Feke et al. (2015) showed that differences in venous blood flow helped to distinguish MCI from AD and healthy control.

Larger population studies suggest that retinal vascular abnormalities are prevalent in AD patients. Using fundus photography and automated vessel segmentation software, Cheung et al. (2014) showed (1) a *reduction* in retinal vessel caliber; (2) a *reduction* in fractal dimension—a measure of the global branching pattern of the retinal vascular tree; (3) an *increase* in vessel tortuosity—a measure of the average degree of curliness/“non-straightness” of retinal vessels; and (4) *no change* to vessel branching angles–the angle subtended between two daughter vessels at each bifurcation. In addition to these parameters, Frost et al. (2013b) also reported that retinal vascular abnormalities, namely venular branching asymmetry and arteriolar length-to-diameter ratios were higher in healthy individuals with high plaque burden, as measured by PET imaging using PiB. Williams et al. (2015) (Williams et al., 2015) reported similar outcome, with the exception of changes in vascular caliber. The authors suggest that static retinal photographs may be more variable due to changes in vascular diameter with the cardiac cycle. Thus, vascular parameters that are robust to pulse driven variability may be useful for screening programs. A challenge in this regard will be whether AD related changes are tractable from age, atherosclerosis and vascular disease related abnormalities. Moreover, whether retinal vascular changes can differentiate mild cognitive impairment from AD is as yet unclear.

### Choroidal thickness

Enhanced depth imaging (EDI) capabilities in modern OCT devices employing longer wavelengths in the range of 1060 nm (Wong et al., 2011) which allows for greater penetration into the deeper layers of the eye, allowing visualization of the choroid. The choroid which sits between the retina and the outer coat of the eye, accounts for the majority of the blood supply to the retina. A number of studies have assessed choroidal thickness changes in AD. Gharbiya et al. (2014) demonstrated that the choroid immediately below and within 1 mm around the fovea was ~30% thinner in AD participants (*n* = 21) compared with healthy controls (*n* = 21). A 20% thinning of the choroid has also been reported by Bulut et al. (2016). Interestingly, Gharbiya et al. (2014) did not find reduced RNFL thickness. While this suggests that choroidal thinning preceded RNFL changes in AD, this should be interpreted with some care given the small sample size, potential noise generated from single-line manual segmentation of the choroid (Cho et al., 2014) and lack of control for signal strength (Ong et al., 2014).

Whilst better segmentation algorithms are already available to overcome the current limitations, the biggest challenge is that choroidal thinning undergoes significant diurnal fluctuations (Kinoshita et al., 2016) and its thinning is seen in many other conditions, including aging (Barteselli et al., 2012), myopia (Ho et al., 2013), uveitis (Baltmr et al., 2014), chronic obstructive pulmonary disease (Ozcimen et al., 2016). It also has limited application in the detection of age-related macular degeneration (Pilotto et al., 2015; Yiu et al., 2015) and glaucoma (Li et al., 2015; Toprak et al., 2016). Again, spatial mapping of choroidal thicknesses in relation to RNFL thinning may reveal specific areas of the eye that are more specific for Alzheimer's disease. This need for specificity underlies studies that have attempted to image hallmark AD deposits in the retina.

### Intraretinal amyloid and tau deposition

In humans, Löffler et al. (1995) provided a histological demonstration that APP and Aβ accumulated (extracellularly, in sub-retinal deposits) in normal aged retinas. It was not known if any of these patients had AD prior to death. It has been known for more than a decade that Aβ is found in drusen deposits in eyes with age-related macular degeneration but not in normal retinas (Johnson et al., 2002; Dentchev et al., 2003; Anderson et al., 2004). More recent findings suggest that Aβ deposits might also occur inside retinal neurons.

Studies in animal models confirmed that APP is expressed by retinal ganglion cells, in the inner nuclear layer of the retina (cell bodies of bipolar, amacrine and horizontal cells) and in the retinal pigment epithelium. These cell classes also express β-secretase and are capable of producing Aβ (for review, see Ratnayaka et al., 2015). In genetically modified mice that have abnormalities in APP processing, Aβ accumulates sporadically within the RGC (Dutescu et al., 2009; Du et al., 2015), in the plexiform layers (Perez et al., 2009), in the nuclear layers (Shimazawa et al., 2008), in blood vessel walls (Liu et al., 2009), Bruch's membrane, outer segments of photoreceptors (Hoh et al., 2010) and within the retinal pigment epithelium (Park et al., 2014). In humans, Hoh et al. (2010) also reported that Aβ accumulated in photoreceptor outer segments in post-mortem retina of AD patients. Given that PET imaging detected amyloid deposition in the brain well before the onset of clinical symptoms in AD (Pike et al., 2007; Rowe et al., 2007), retinal imaging of *in vivo* Aβ accumulation is an exciting prospect.

#### Tagging AD deposits

Koronyo-Hamaoui et al. (2011) were first to demonstrate successful non-invasive *in vivo* visualization of curcumin bound flurorescent Aβ in the retinas of bitransgenic mice (APP_swe_/PS1_ΔE9_), with mutations to genes that regulate APP and PS-1. They verified these findings using immunohistochemistry co-labeling via anti-Aβ specific antibodies and Thioflavin-S. By vaccinating with an altered myelin-derived peptide (MOG45D), which has been shown to reduce Aβ plaques in the brain (Koronyo-Hamaoui et al., 2009), the authors showed that retinal deposits behave in a similar way, with fewer and smaller Aβ plaques. Specific curcumin binding of Aβ has also been demonstrated in donor brains and retinas from AD patients. No such staining was seen in healthy controls of the same age. The Commonwealth Scientific and Industrial Research Organization (CSIRO, Australia) have shown promising preliminary data in humans employing curcumin contrast in patients confirmed to have deposits in the brain using PiB-PET imaging (Frost et al., 2014).

Though promising, other studies have not found the same outcome. Schön et al. (2012) in a small sample of 6 post-mortem retinas from confirmed AD patients failed to find Aβ plaques. They also report that they were unable to detect Aβ plaques *in vivo* using amyloid-binding fluorophores (BSc4090) in APP_swe_/PS1_ΔE9_ mice. Schön et al. (2012) suggest that hyperphosphorylated tau could potentially be a better marker for the disease, having demonstrated its presence in the retina of the P301S human tau mouse line using *in vivo* scanning laser ophthalmoscopy imaging of (trans,trans)-1-fluoro-2,5-bis(3-hydroxycarbonyl-4-hydroxy)styrylbenzene (FSB)-bound tau aggregates. These tau aggregates were also present in 5 out of 6 of the AD human retinas studied. However, Ho et al. (2014) failed to find either phospho-tau deposits or Aβ in the retina of 11 confirmed AD donors using standard histological techniques, including Aβ antibodies, thioflavin and congo red, that are well established for brain tissue. The authors conclude that AD hallmarks do not deposit in the eye in a manner analogous to the brain, though they concede that the use of different assays; limited retinal cross sections instead of wholemount; and paraffin embedded sections instead of frozen tissues; might limit the sensitivity in the detection of these proteins.

#### Visualizing AD deposits without tagging

Newer imaging modalities that target specific reflectance characteristics of biological material hold the promise of being non-invasive, contrast agent free and potentially specific. One such method is hyperspectral imaging, whereby tissue reflectance to a wide range of incident wavelengths are quantified (More and Vince, 2015). Using this technique, More and Vince (2015) were able to determine that Aβ has a unique hyperspectral signature, capable of distinguishing these deposits in *ex vivo* preparations of brain and retina from a mouse model of AD. More recently, the authors applied this technology to a live mouse retina, demonstrating that the hyperspectral signatures are preserved even when imaged through the ocular media. This discovery provides the first evidence for the non-invasive *in vivo* detection of AD using hyperspectral imaging without the need for an extraneous agent (More et al., 2016).

Another emerging imaging technique is the use of cross-polarizers to distinguish *ex vivo* human donor retinas with AD from controls (Campbell et al., 2015; Hamel et al., 2016). This method capitalizes on the fibrillary arrangement of Aβ resulting in specific changes to birefringence that are quantifiable using Mueller matrix polarimetry. Along these lines, the advent of PS-OCT may also prove to be useful.

Direct visualization of AD hallmarks with or without a contrast enhancing agent in the retina may be considered the most promising biomarker due to its specificity for the disease. However, ongoing work is needed to verify that Aβ plaques or accumulations, are indeed present in human retinal tissues; and that such deposits indicate or are predictive of brain deposits.

### Electrophysiological signature

Functional change in the electrical response of specific brain regions may be early markers in AD. This might arise as neuronal changes signaling damage which may precede the conversion to clinical disease (Alberdi et al., 2016). Not surprisingly, researchers have measured visually evoked potentials, using electrodes on scalp overlying the occipital region on either side of inion, as a way to differentiate AD and healthy control volunteers. A checkerboard stimulus that is reversing in contrast produces P1 and N1 components that are substantially reduced in patients with advanced AD (Parisi et al., 2001; Stothart et al., 2015). This is associated with losses in the retinal ganglion cell derived pattern electroretinogram, which is measured using electrodes on the eye in response to the same stimulus (Parisi et al., 2001; Stothart et al., 2015). Furthermore, studies using multifocal electroretinogram, which uses finer checks to allow spatial localization of responses to small patches of retina, have shown changes in the macular region in early AD patients (Moschos et al., 2012). Electroretinogram deficits have also been found in murine models of Aβ deposition (Perez et al., 2009; Krantic and Torriglia, 2014). More recently, Parthasarathy et al. (2015) and Gupta et al. (2016) showed an association between Aβ burden in the retina and inner retinal function; suggesting altered neurotransmission between photoreceptors and inner retinal cells. Whether such changes are specific to AD remains to be seen.

## Non-retinal biomarkers

### Pupillary reactions

Pupil size and the pupillary response to light is determined by the balance of forces exerted by the iris sphincter and dilator muscles. The former is influenced by cholinergic receptors found in the parasympathetic system originating from the Edinger-Westphal nucleus; whilst the latter is innervated by noradrenaline receptor based postganglionic sympathetic system arising from the superior cervical ganglion. It is well established in AD that selective nicotinic acetylcholine receptor loss accounts for symptoms closely associated with the severity of the disease (Buckingham et al., 2009). Indeed, four out of the five US Food and Drug Administration approved treatments for AD are cholinesterase inhibitors, which promote neurotransmission and masks some of the symptoms of AD.

Scinto et al. (1994) proposed the use of a clinical “pupil dilation test” to expose AD cholinergic dysfunction. Central cholinergic depletion results in upregulation of peripheral receptors and thus hypersensitivity to cholinergic agonists, or a reduced sensitivity to cholinergic antagonists. Using a dilute agonist such as pilocarpine 0.0625% (Idiaquez et al., 1994) or muscarinic antagonist such as tropicamide 0.01% induced an abnormally large pupillary constriction or dilation in AD patients, respectively. An alternative explanation was offered by Hou et al. (2006) who showed evidence that the locus coeruleus of the brain was responsible for adrenergic iris dilator input; which when damaged in AD resulted in less sympathetic monotone resistance to low dose tropicamide in AD. Recent studies have been unable to replicate these findings. In a review of more than 20 available studies on the subject, Frost et al. (2010) concluded that the reliability of a “pupil dilation test” for AD remained questionable.

Given that cholinergic neurotransmission is altered in AD, this might result in a change in the pupil's response to light. Indeed, a decrease in pupil diameter response to abrupt changes in room illumination (Prettyman et al., 1997) or a single flash of light (known as the pupil flash response, PFR) has shown promise as a biomarker for AD (Fotiou et al., 2000). The latter study went on to show that the PFR parameter that best distinguished AD patients from healthy controls was the maximum constriction acceleration (Fotiou et al., 2007). Others have also linked PFR changes to amyloid plaque burden in the brain (Frost et al., 2013) and were also able to distinguish asymptomatic carriers of an amyloid beta gene mutation (APPGlu693Gln) from non-carriers (Frost et al., 2013). Whilst standard flash pupillometry was able to distinguish AD from healthy controls, only subtle timing differences were reported between groups.

Modifications are therefore being developed to enhance the sensitivity of this technique such as the use of chromatic light to target specific susceptible neurons in the retina, in particular the retinal ganglion cells (Rukmini et al., 2015). Another possibility is the use of a slower flash with an upwards “ramp” of increasing intensity may draw out more subtle differences in maximum constriction acceleration (Lei et al., 2014).

To this end, Bittner et al. (2014) suggest that repetitive flashes fatigue the system resulting in more pronounced PFR amplitude attenuation in AD and MCI patients compared with healthy controls, and correlated better with cognitive test scores (MMSE). They hypothesized that repeated light stimulation in AD patients challenges the interaction between a deficient sympathetic tone and parasympathetic overtone, resulting in smaller pupils and progressive loss of amplitude. Taken together, these studies show that pupil flash responses using high-speed video-pupillography might be a promising biomarker as it is directly assesses cholinergic integrity in the nervous system. It remains to be seen whether taking AD medications such as cholinesterase inhibitors blunt this effect. Certainly, more studies are required to establish the reliability and repeatability of this emerging technology.

### Imaging the crystalline lens

The role of the crystalline lens is to focus incident light from the outside world onto the retina. Primarily composed of crystallin proteins, the high protein concentration and clear optics presents an opportunity for researchers to study protein aggregation in the eye. Aβ has been shown to deposit in the crystalline lenses of monkeys, rats, rabbits (Frederikse et al., 1996) and transgenic mice (Frederikse and Ren, 2002). Goldstein et al. (2003) first reported the discovery of an AD specific cataract in humans, where they show Aβ deposits in the cytoplasm of the equatorial supranuclear/deep cortical lens fiber cells. This “AD cataract” does not impair vision and is difficult to detect in a routine eye examination unless a full dilation is achieved (Moncaster et al., 2010). Others were unable to detect Aβ in the lens using either standard immunohistochemistry methods (Michael et al., 2013; Ho et al., 2014; ) or confocal Raman spectroscopy (Michael et al., 2014). In a clinical study, Bei et al. (2015) did not find specific cataracts in a cohort of AD patients whose pathology have been confirmed using both PiB-PET imaging and CSF analysis.

Kerbage et al. (2013) suggested using a florescent amyloid binding ligand in order to maximize the chances of detecting Aβ in the lens. By compounding the ligand substance (aftobetin hydrochloride) into a sterile ophthalmic ointment suitable for topical application; in combination with an *in vivo* pulsed laser fluorescent spectroscopy in a group of 20 AD and 20 healthy controls the authors were able to detect supranuclear amyloid in the lens of most AD patients with a sensitivity of 85% and specificity of 95% (Kerbage et al., 2015). The authors found a correlation between fluorescence uptake values in the lens with amyloid burden in the brain detected quantified using PET.

**Figure 5:**
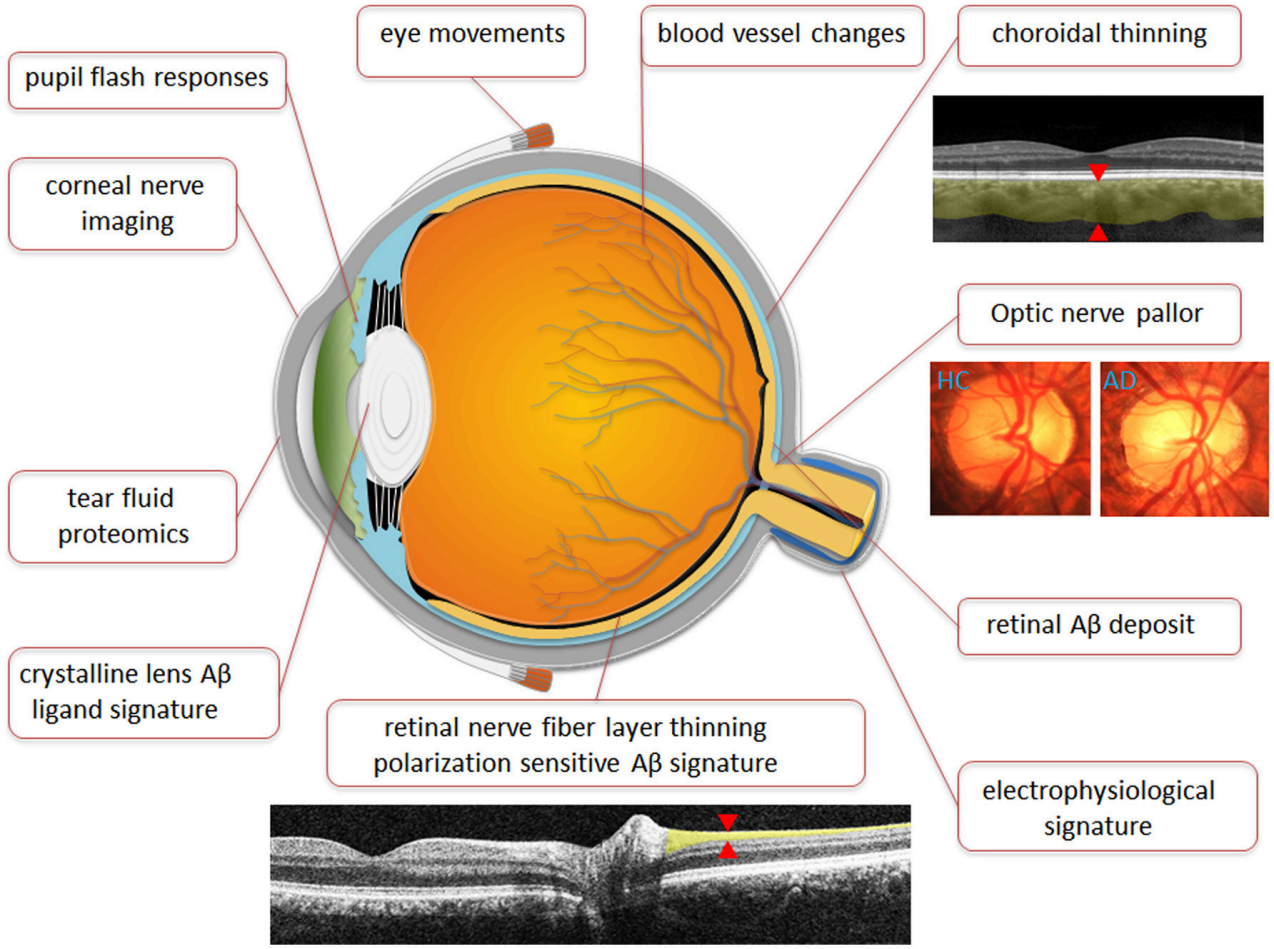
**Anatomical representation of Ocular biomarkers**. Figure illustrates ocular biomarkers which have shown changes in AD patients as well as potential future ocular biomarkers as marked by location in the eye. Images are broadly representative of the techniques employed in studies. Red arrows denote where measurements are typically taken.

Despite the promise of Kerbage et al.'s (2013) recent study as a whole, the literature indicates that the presence of Aβ in the human lens remains to be confirmed. Nevertheless, it appears that fluorescently labeling Aβ in the lens may provide improved detection over conventional lenticular assessment. Further studies are needed to establish this is the case in larger cohorts of patients. Furthermore, ongoing studies are needed to prove that the system is capable of not just detection but potentially be able to stratify patients based on disease severity. Table 2 summarizes the key studies which have shown promise as utility of the eye as a biomarker for AD. Figure 5 schematically highlights those sites in the eye which show evidence for changes with AD as well as those currently being further investigated.

**Table 2:** **Ocular changes associated with Alzheimer's disease**.

**Structure**	**Biomarker**	**Highlights**
Pupils	Pupil flash response^a^	High speed video pupillography Reduced mean constriction velocity sensitivity 0.74, specificity 0.71, AUC 0.76 Reduced maximum constriction, AUC 1.0 Constriction amplitude decreased in AD with repeated flashing
Lens	Aβ accumulation in supranuclear layer^b^	Topical Aftobetin hydrochloride ointment with fluorescent ligand eye scanning system shows greater fluorescence in AD vs. HC. sensitivity 0.85, specificity 0.95, AUC 0.915
Retina	Aβ hallmark detection^c^	Curcumin (injected and/or orally administered) binding to amyloid in mouse retina, fundus camera. Hyperspectral analysis distinguishes normal from AD mice. Amyloid fibrillary signature detected using Mueller matrix polarimetry.
	OCT^d^	Retinal nerve fiber layer thinning Superior RNFL, AUC 0.60 Macula Ganglion Cell complex, AUC 0.66 Choroidal layer thinning using EDI-OCT
	Vascular changes^e^	Retinal venous blood flow reduction Retinal vessel width reduction Retinal vessel increased tortuosity Retinal vessel increased branching complexity Pattern Electroretinogram: Slower N35, P50 implicit time and reduced P50 and N95 amplitudes Multifocal Electroretinogram: reduced P1 amplitudes Pattern Visual Evoked Potential: Slower P100 implicit time
	Electroretinogram Visual Evoked Potential^f^	
Optic Nerve	Pallor^g^	Optic disk color analyzed using Laguna Optic Nerve Hemoglobin software shows reduced optic nerve hemoglobin in AD.

*This table shows a summary of key studies documenting ocular manifestations in Alzheimer's disease*.

a *Prettyman et al. (1997), Fotiou et al. (2000), Fotiou et al. (2007), Frost et al. (2013a), Bittner et al. (2014)*.

b *Kerbage et al. (2013, 2015)*.

c *Koronyo-Hamaoui et al. (2011), Campbell et al. (2015), More and Vince (2015)*.

d *Parisi et al. (2001), Iseri et al. (2006), Berisha et al. (2007), Paquet et al. (2007), Lu et al. (2010), Kesler et al. (2011), Kirbas et al. (2013), Moreno-Ramos et al. (2013), Ascaso et al. (2014), Larrosa et al. (2014), Cheung et al. (2015)*.

e *Berisha et al. (2007), de Jong et al. (2011), Cheung et al. (2014), Frost et al. (2013b), Feke et al. (2015), Williams et al. (2015)*.

f Parisi et al. (2001) Krasodomska et al. (2010), Sartucci et al. (2010), Moschos et al. (2012), Stothart et al. (2015)

g *Tsai et al. (1991), Bambo et al. (2015)*.

### Eye movements

It has been recognized that patients with AD suffer difficulty with reading. This is in part due to suboptimal eye movements, which has been suggested to be linked to memory function (Fernández et al., 2014). It has been shown that AD sufferers present with increased latency when initializing voluntary eye movements, show decreased eye movement velocity, fail to fixate on a target or move in the wrong direction entirely and also fail to follow a moving target (Molitor et al., 2015). These fixation and movement errors reflect damage to their neural generators within the cortex and the brainstem. Although researchers have yet to find a distinct AD signature, eye movements show potential as a biomarker. Further studies are needed to establish a standardized test battery specific to AD and to demonstrate the ability to correlate oculomotor function with disease progression.

## Potential future biomarkers

### Corneal nerve imaging

Corneal nerves are stratified into 3 layers and may be found in between the basal epithelium and bowman's layers (sub-basal nerve plexus), in between the bowman's layer and the anterior stroma (sub-epithelial nerve plexus) and within the stroma (stromal nerve plexus). Corneal nerve imaging has clinical utility post-corneal surgery as well as in diseased state such as keratoconus (a corneal ectasia), dry eyes and in diabetes (Patel and McGhee, 2009). Nerve growth factor is essential for the development and maintenance of neurons in the peripheral nervous system and for the integrity of cholinergic neurons in the central nervous system and in the cornea. As impaired nerve growth factor retrograde transport has been linked to cholinergic neurodegeneration in AD (Aloe et al., 2012) corneal nerve integrity might prove useful as a biomarker for the disease. Preliminary steps are being taken to be able to image axonal transport *in vivo* (Abbott et al., 2013; Takihara et al., 2015). As corneal nerves can be imaged directly, a functional measure of axonal transport may prove to be more specific for AD.

### Ocular fluid biomarkers

Proteomics have been increasingly used since the 1990's for large scale analysis of proteins in health and diseases, such as AD (Butterfield, 2004). Proteomic analyses of aqueous humor samples have revealed the presence of AD related peptides. Tear fluid, being easy to access has also shown promise in the detection of neurodegenerative diseases such as glaucoma (Tezel, 2013) and was recently shown to be clinically viable (Kalló et al., 2016).

Micro RNA (miRNA) has gained significant attention as a class of non-coding regulatory RNA molecules capable of modifying gene expression at the post-transcriptional level by binding to the un-translated region of their target mRNAs. Several candidate miRNA namely hsa-miR-106, −153, −101, −29, −107 have already been shown to be implicated in regulating amyloid production (Kumar et al., 2013). That miRNA molecules have been isolated in tears warrants further studies as to their feasibility as a fluid biomarker for AD.

## Summary

Only five treatments are currently approved by the United States Food and Drug Administration for AD, namely, rivastigmine, galantamine, tacrine, donepril and memantine. These are all cholinesterase inhibitors with the exception of memantine, which is an NMDA (N-methyl-D-aspartate) receptor antagonist. These medications delay the worsening of the symptoms in AD for some 6 to 12 months and work in about half of patients (Casey et al., 2010). As yet there is no approved treatment targeting the pathophysiological mechanisms underlying AD. Current candidate AD drugs include those that target either Aβ, APP, or tau metabolism by preventing oligomer efflux, modulating enzyme secretases, prevent aggregation, facilitate clearance and vaccination induced immunological clearance (Kumar et al., 2015). To test such drugs reliable and sensitive laboratory and clinical readouts are needed.

The discovery of AD biomarkers such as PET imaging and CSF molecules have considerably advanced our understanding of the disease. These biomarkers are crucial for disease monitoring and the recruitment of preclinical AD patients for clinical trials. Despite the success of these established biomarkers, their widespread implementation remains a challenge. Ocular biomarkers for AD are still in their infancy and are not without their limitations. Many of the potential biomarkers discussed share substantial overlap with ocular and systemic diseases. Currently, the ocular biomarkers holding the most promise are those specific for AD pathophysiological such as the detection of Aβ-related retina changes. Additionally or alternatively, a battery of clinical functional and structural ocular assessments may improve specificity for AD. Eye care practices are well positioned to provide these technologies to the public. Already, sensitive imaging modalities such as fundus cameras and OCT are commonplace in eye clinics. Emerging technologies such as OCT angiography (using OCT to resolve the smallest blood vessels), EDI-OCT and PS-OCT will contribute to the increase in sensitivity and diagnostic capacity for the disease. With advances in this area, primary eye care practitioners may play a larger role in the provision of care for patients with Alzheimer's disease. A range of neurological diseases including AD, Parkinson's disease, frontotemporal dementia, vascular dementia, Neimann-Pick disease may stand to benefit from ocular biomarker technology, as a means to improve understanding, monitoring and to help facilitate of discovery of therapies.

## Author contributions

JL, BB, CN have made the following author contributions: Substantial contributions to the conception or design of the work; Drafting the work and revising it critically for important intellectual content; Final approval of the version to be published; Agreement to be accountable for all aspects of the work in ensuring that questions related to the accuracy or integrity of any part of the work are appropriately investigated and resolved. QL, ZH, AV, VW, NC, JM have made the following author contributions: Substantial contributions to the conception or design of the work; and Revising the work critically for important intellectual content; Final approval of the version to be published; Agreement to be accountable for all aspects of the work in ensuring that questions related to the accuracy or integrity of any part of the work are appropriately investigated and resolved.

## Funding

CN, BB, AV, JM, (all authors), Samantha Budd Haeberlein, are joint investigators on an Australian Research Council Linkage grant LP160100126. BB is a sole investigator on Australian Research Council Linkage grant FT130100338.

### Conflict of interest statement

CN, BB, AV and JM are joint investigators on an Australian Research Council Linkage grant LP160100126. NC is an employer of Biogen Inc. JM is an employee of AstraZeneca Neuroscience. All other authors declare that the research was conducted in the absence of any commercial or financial relationships that could be construed as a potential conflict of interest.
